# The inhibitory effect of a novel neem paste against cariogenic bacteria

**DOI:** 10.4317/jced.58781

**Published:** 2021-11-01

**Authors:** Tasanarong Tasanarong, Somying Patntirapong, Visakha Aupaphong

**Affiliations:** 1 Faculty of Dentistry, Thammasat University, Rangsit campus, Pathum Thani, Thailand

## Abstract

**Background:**

Dental caries is a major oral health problem, which associates with cariogenic bacteria. *Streptococcus mutans* and *Lactobacillus acidophilus* are facultative anaerobic bacteria that are found in tooth decay. Accordingly, neem leaf extract was developed due to its great anti-microbial property against many bacteria. The aim of this study was to determine anti-cariogenic properties of neem leaf extract in a novel paste preparation.

**Material and Methods:**

The neem extract was derived from maceration of dry neem leaves in ethanol for 48 h. The ethanolic extract was subjected to chemical identification using GC-MS. Neem pastes were prepared from ethanolic extract mixed with polyethylene glycol paste with or without zinc oxide. *S. mutans* and *L. acidophilus* test were initiated at bacterial concentration of 108 CFU/ml. The antibacterial activity was then performed by disc diffusion method following by minimum bactericidal concentration (MBC) technique.

**Results:**

GC-MS result displayed 35 compounds. Compounds found in the extract were n-Hexadecanoic acid (31.18%), Hentriacontane (18%), Phytol (16.79%). Disc diffusion showed that ethanolic extract and neem pastes inhibited growth of both bacteria. For MBC, neem paste with zinc oxide at concentration of neem 0.4 mg/ml was the most effective concentration on inhibiting *S. mutans* growth. Neem pastes and ethanolic extract at concentration of neem 6.25 mg/ml inhibited *L. acidophilus* growth.

**Conclusions:**

The ethanolic neem leaf extract and novel neem pastes had antimicrobial effect on both *S. mutans* and *L. acidophilus*. By this property, neem paste could be developed for the application in dental field, i.e. pulp capping.

** Key words:**Neem, Azadirachta indica, antimicrobial, cariogenic bacteria.

## Introduction

Dental caries is a health issue affecting people worldwide. It has been estimated in Asian population around 52.6 % in primary teeth and 58.8% in permanent teeth (1). Dental caries is caused by multiple factors, which is involved specific types of microorganism. Dental caries process starts from demineralization of the inorganic component and subsequently degradation of the organic substance. When left untreated, caries will progress into the pulp tissue and eventually the tooth will be lost. Managing deep carious lesions to avoid pulp exposure and root canal treatment is considered being the cost-effective treatment (1-3).

A technique in dental restorations, called pulp capping, is used to prevent the dental pulp from necrosis after being exposed or nearly exposed during a cavity preparation. This technique is beneficial for the success of vital pulp therapy because it can protect the pulp against external irritants such as microbial challenges, resulting in a long-term survival of pulp tissue (4,5). One of the ideal properties of pulp capping material should include bactericidal or bacteriostatic effect (6). Nowadays, calcium hydroxide (Ca(OH)2) has been accepted as a standard pulp capping material because it has excellent antibacterial result (7). However, it has disadvantages that need to be considered. Ca(OH)2 is highly soluble, lacks adhesive property, has high pH (8), produces multiple tunnel defect in dentine bridge (9,10), dissolves odontoblast layer (11), and induces pulpal inflammation for up to 3 months (12).

Neem (*Azadirachta indica*) is typically grown in tropical and semi-tropical regions such as in Thailand and India. Its leaf, bark, and seed are used for therapeutic purposes. Numerous biological and pharmacological activities of neem leaf has been reported including antibacterial (13), antifungal (14), and anti-inflammatory activities (15). Neem leaf extract potently inhibits many bacteria such as *S. mutans*, *Enterococcus faecalis*, and **Candida* albicans* in biofilm (16), *Lactobacillus spp*. (17). Neem leaf extract also has effect on proinflammatory cell signaling (18). From previous studies, neem leaf has many suitable properties to develop as a cavity liner to preserve dental pulp tissue, which can be affected by carious products.

Neem inhibits many bacterial growth, however, the mixture of neem and paste has not been developed and tested. Thus, this experiment aimed to test the anti-cariogenic properties of neem leaf extract in a novel preparation using neem extract mixed with polyethylene glycol paste. *S. mutans* and *L. acidophilus* were evaluated because these two bacteria are considered to be the most important cariogenic bacteria associated with dental caries.

## Material and Methods

-Neem extraction 

Mature fresh neem leaves were collected form a farm in Thailand. The neem sample was sent for taxonomic identification at Princess Sirindhorn Plant Herbarium. The sample was identified as *Azadirachta indica* A. Juss. var. *Siamensis* Valeton. Fresh neem leaves were washed under tap water to eliminate dust and foreign particles, dried under the shade for 4 h, and then dried under oven at 50°C for 1 h. The dried leaves were crushed into neem powder. Neem powder at 30 g were extracted with 95% ethanol, methanol or distilled water. The solutions were incubated in shaker incubator for 24 h. Then, the mixture was boiled in hot water bath for 30 min and further incubated in shaker incubator for 24 h. After incubating, the solution was filtered using Whatman No.1 filter paper with vacuum. The organic solvents were evaporated by rotary evaporator under reduced pressure at 40°C to obtain crude extract. For the aqueous extract, the solvent was evaporated by freeze dry machine.

-GC-MS analysis 

Ethanolic extract was done to identify constituents in the extract using gas chromatography-mass spectrometry (GC-MS). GC-MS was performed at the Office of Advanced Science and Technology, Thammasat University. Ethanolic extract was subject to GC by 7890B, Agilent technology, USA, and MS by 55977A, Agilent technology, USA, auto-sampler model. For GC, helium was used as carrier gas at a constant flow of 1 ml/min through HP-Innowax column. An injection volume of 1.0 µl was employed (split ratio of 20:1) injector at a temperature of 250°C. The temperature was programmed with an increase of 8°C/min from 50°C to 250°C, and then held for 15 min at 250°C. For MS, the electron impact was run at 70 eV with full scan mode and at mass range 45-700 m/z. at 40 min.

-Paste preparation

Six paste forms were prepared. Each preparation contained different composition of zinc oxide and concentration of neem extract. The paste base included polyethylene glycol, stearyl alcohol, glycerol, sodium lauryl sulfate, and distilled water. Paste 1 (P1) was paste base without zinc oxide. Paste 2 (P2) was 25 mg/g neem in paste base without zinc oxide. Paste 3 (P3) was 100 mg/g neem in paste base without zinc oxide. Paste 4 (P4) was paste base with zinc oxide. Paste 5 (P5) was 25 mg/g neem in paste base with zinc oxide. Paste 6 (P6) was 100 mg/g neem in paste base with zinc oxide. Pastes were prepared to a final concentration of 20 mg/ml in water. Then, pH of the preparations was evaluated using a pH meter (Thermo Sciencetific Orion Versastar pro). The measurement was carried out in triplicates.

-Antibacterial activity

•Disc diffusion

The study of antimicrobial effect of the extractions and neem paste using agar disc diffusion method. The *S. mutans* (ATCC25175) in Müeller-Hinton broth at 0.5-0.7 OD using an Ultraspec 2000 spectrophotometer at 600 nm were used in this experiment (108 CFU/ml). The 200 µl of bacteria suspension was placed on Mitis salivarius agar surface. The *L. acidophilus* (ATCC4356) in de Man Rogosa and Sharpe (MRS) broth at the similar OD and volume also used and placed in MRS agar surface. The methanolic extract, ethanolic extract, aqueous extract (25 mg/ml), 0.2% chlorhexidine, Ca(OH2) paste, 0.9% Normal saline (NSS), P1, P2 , P4 and P5 were examined by immersing 6 mm paper disc into 30 µl of solution or 1.5 mg of paste. Then, they were incubated with gas pack at 37°C for 48 h. The diameter of inhibition zones was measured and recorded in centimetre (cm). The experiments were done in 5 independent experiments and each experiment was done in triplicate.

•Minimum Bactericidal Concentration (MBC) 

The concentrations of P1 and P4 were five-fold serial dilutions ranging from 1.6 to 1000 mg/ml. The ethanolic extract was two-fold serial dilutions ranging from 0.2 to 25 mg/ml. P3 and P6 were two-fold serial dilutions concentration of neem ranging from 0.4 to 6.25 mg/ml. The 100 μl of each solution was placed into a sterile 96-well microtiter plates. The 100 μl of bacterial inoculum (108 CFU/ml) were added to each well. The covered microtiter plates were incubated with gas pack for 24 h at 37°C. Then, 100 µl aliquot from wells were cultured on 60×15 mm nutrient agar plates to determine the bactericidal activity, and incubated with gas pack for 48 h at 37°C.

-Statistical analysis

All data was determined for normal distribution using Kolmogorov–Smirnov normality test. The differences among groups were calculated by One-way ANOVA with Tukey’s multiple comparison test. The data was presented by means ± SE. Significance was assigned as *p<0.05, ** *p*<0.01, *** *p*<0.001 vs. 0.2% chlorhexidine, # *p*<0.05, ## *p*<0.01, ### *p*<0.001 vs NSS, and 0 *p*<0.05, 00 *p*<0.01, 000 *p*<0.001 vs Ca(OH)2.

## Results

-GC-MS

**Figure 1:**
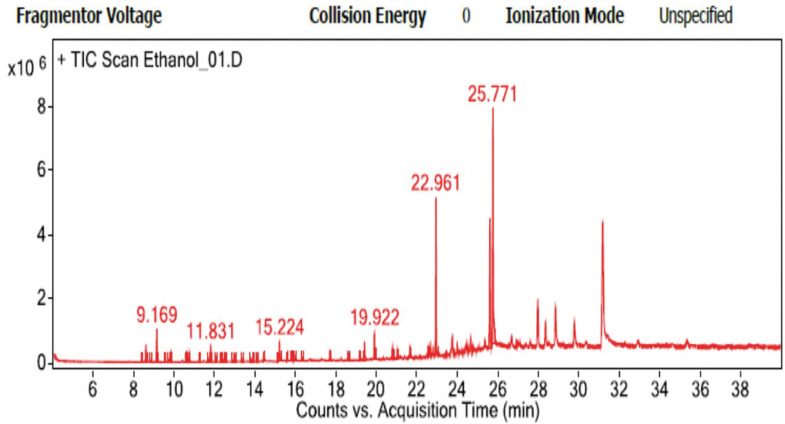
GC-MS chromatogram of ethanolic neem leaf extract.

GC-MS result displayed 35 compounds. n-Hexadecanoic acid was most be found in this extract at RT 25.771 (31.18%), Hentriacontane at RT 25.63 (18%), Phytol at RT 22.961 (16.79%), N-methyl-N-(methyl-d3) aminoheptane at RT 19.922 (3.2%), Neophytadine at RT 15.224 (2.87%), alpha-Copaene at RT 9.169 (3.34%), alpha-cubebene at RT 8.63 (1.87%), alpha.-Humulene at RT 11.83 (1.7%), and other miscellaneous compounds (15.05%), respectively (Fig. 1).

-Antibacterial activity

**Figure 2:**
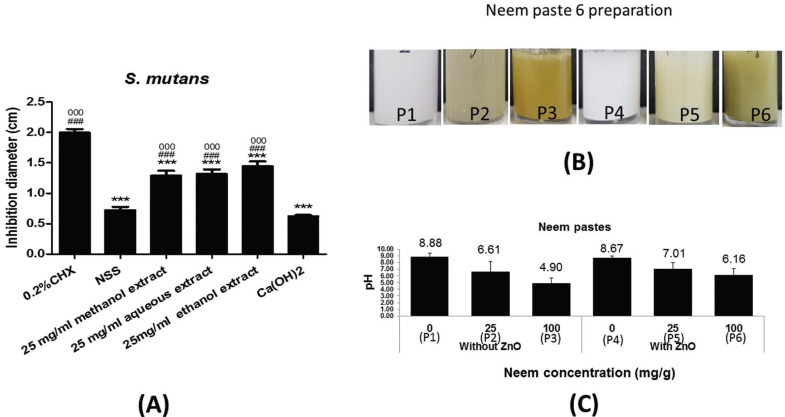
A) Inhibitory effects of three neem leaf extract solvents on *S. mutans* growth. B) The soluble property of the preparations. P1; paste without zinc oxide, P2; 25 mg/g neem paste without zinc oxide, P3; 100 mg/g neem paste without zinc oxide, P4; paste with zinc oxide, P5; 25 mg/g neem paste with zinc oxide, P6; 100 mg/g neem paste with zinc oxide. The mixture of neem pastes showed variety of brownish green color. (C) Graph demonstrated pH of neem paste preparations.

In order to screen for suitable solvent for neem extraction, the neem leaves were extracted by three different solvents and then tested antimicrobial activity on *S. mutans*. Chlorhexidine (0.2%), normal saline (NSS) and Ca(OH)2 were served as positive control, negative control, standard treatment, respectively. All three extracts showed significantly higher inhibition zone than NSS and Ca(OH)2. The ethanolic extract yield a better inhibitory effect by up to 2.3-fold of Ca(OH)2. When compared with chlorhexidine, all extracts were lower in inhibition zone. There was no significant difference among all three extracts tested (Fig. 2A). However, the ethanolic extract seemed to have better impact on bacterial growth inhibition. Therefore, ethanolic extract was the first option for neem paste preparation used later in this study.

After obtaining an appropriate solvent, the neem leaves were extracted by ethanol and prepared in paste form. Then, we tested the soluble property of neem pastes. All preparations were able to dissolve in water in a miscible-liked consistency. Next, the pH of each preparation was examined. The pH of paste P1 and P4 were approximately 8.9 and 8.7, respectively. Addition of ethanolic neem extract in the paste decreased the pH in a dose-dependent manner. The peak reduction of pH was seen in neem paste without zinc oxide at concentration 100 mg/g (Fig. 2B,C).

**Figure 3:**
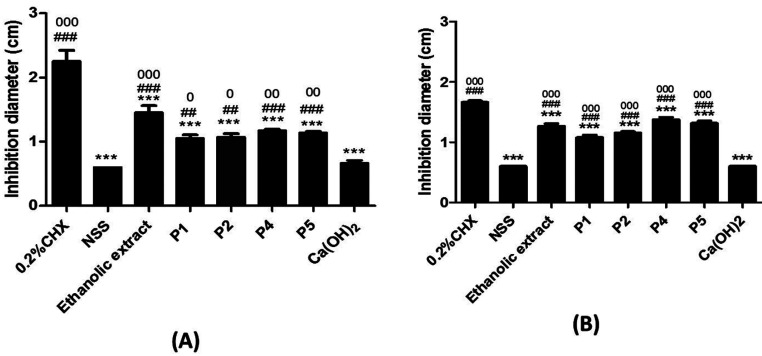
The effect of neem pastes on tested microorganisms. A) *S. mutans*. B) *L. acidophilus*.

Neem pastes were investigated for their antibacterial activity against *S. mutans* and *L. acidophilus* by disc diffusion method. All preparations inhibited both bacterial growth when compared with NSS and Ca(OH)2. Furthermore, ethanolic extract was able to increase inhibition diameter more than P1, NSS and Ca(OH)2. No significant difference was observed among ethanolic extract, P2, P4, and P5. However, the effect of all samples was not on the level of chlorhexidine (Fig. 3).

**Figure 4:**
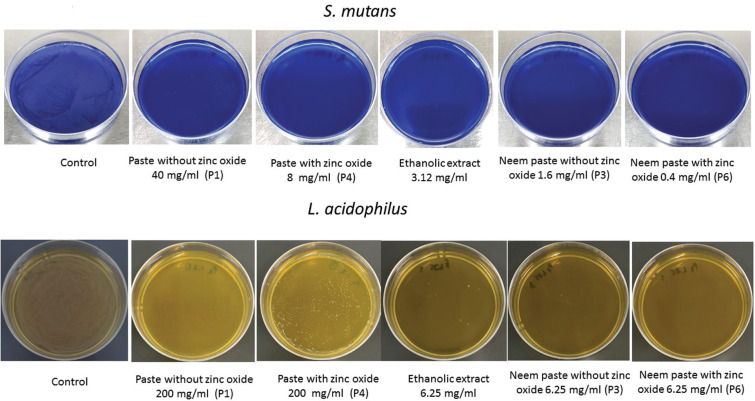
The effect of neem pastes on tested microorganisms. A) *S. mutans*. B) *L. acidophilus*.

In case of *S. mutans*, MBC was 40 mg/ml for P1, 8 mg/ml for P4, 3.12 mg/ml for ethanolic extract, 1.6 mg/ml for P3, and 0.4 mg/ml for P6. In case of *L. acidophilus*, MBC was 200 mg/ml for P1, 200 mg/ml P4, 6.25 mg/ml for ethanolic extract, 6.25 mg/ml for P3, and 6.25 mg/ml for P6. (Fig. 4).

## Discussion

One of the most common oral health problem is dental caries. Acidogenic and aciduric gram-positive bacteria, primarily *S. mutans* and *lactobacilli*, cause dental caries. Since many component of *A. indica* exhibit antimicrobial characteristics, we investigated the antibacterial activities of neem leaf extract in paste form against selected oral cariogenic microorganisms. This study showed that all preparations were able to successfully eradicate certain bacterial strains.

Neem extract obtained by ethanolic agent was more effective in inhibiting bacteria than those obtained by aqueous and methanolic agents. The ethanolic extract produced a number of constituents than aqueous extract, which was in accordant to previous study by Shewale and Rathod (19). This data suggested that neem extracted by ethanol was the optimal condition.

The pastes had a pH of 4.9 to 8.8, indicating that they were acid and mild alkaline. It has been shown that *S. mutans* and *L. acidophilus* are well function or growth within the pH range of 3 – 6 (20,21). Therefore, it is possible that pH of paste with zinc and paste without zinc (8.7 and 8.9) could inhibit specific bacterial growth such as *S. mutans* and *lactobacilli*. Additionally, polyethylene glycol in the paste inhibits multiplication and development of bacteria by removal of water (22,23). Also zinc oxide inhibits enzyme metabolism of bacteria (24).

However, the addition of neem extract in the pastes lowered the pH in a dose-dependent manner. This showed that neem paste can be acidic depending on the percentage of the leaf extraction. The pH dropped to 4.9 – 6.1, which is the range that bacteria can survive. It could be possible that constituents in the paste and neem extract would be the factors inhibiting bacterial growth. GC-MS method yielded many constituents in neem. Phytol, which is a diterpenes, was found to be around 16.8%. Phytol (RT 22.961 in GC-MS graph) can decrease the level of bacterial counts *in vivo* (25). Dodecanoic acid or lauric acid (RT 21.677 in GC-MS graph), a type of medium-chain fatty acids, was also obtained but in a small amount. This constituent reduces biofilm formation *in vitro* (26) and restrains oral bacterial growth (27).

Other medicinal herbs that have anti-microbial activity also contain substances found in neem extract. This includes such as β-Bourbonene (28), β-copaene, β-caryophyllene (29), δ-Cadinene (30), Neophytadiene (31). Moreover, some substances in neem, such as γ-Muurolene, has anti-nociception property in tested animal models (32). However, these isolated compounds should be evaluated for their properties in the future.

## Conclusions

The neem paste had antimicrobial effect on *S. mutans* and *L. acidophilus*. This was synergistic effect between constituents in paste and ethanolic extract. The antimicrobial effect could be later applied in pulp capping material.
